# New products

**DOI:** 10.1155/S1463924600000079

**Published:** 2000


Journal of Automated Methods & Management in Chemistry, Vol. 22, No. 2 (March–April 2000) pp. 53–64

### New products

### Intelligent olfaction detection

The QMB 6 intelligent chemosensory system, used with the Perkin-Elmer HS40 XL Headspace analyser, offers fully automated analysis of odorous compounds.

The system consists of six sensor elements coated with different gas-sensitive materials, each oscillating at a 10 MHz frequency. Adsorption of volatiles causes frequency changes, and the resulting signal pattern is evaluated by artificial neural network software, using pattern recognition methods. The system can be 'taught' to recognize specific patterns, as desirable or undesirable, and to reject 'unknowns'.

It is particularly appropriate for quality control in food and aroma analyses, to detect off-odours, or contamination in organic materials and pharmaceutical products, and to detect nuisance smells or harmful substances in the environment.

Application outlines available from Perkin-Elmer illustrate the use of the QMB 6:

- to discriminate plastic materials used in cars;
- to intensify spice mixtures with differing proportions of pepper;
- in the analysis of shampoo concentrates;
- to identify different hop varieties;
- to classify the variety of carrots;
- to aid in classifying solid compost samples.

*For further information contact Carol-Anne Green, Perkin-Elmer Limited, Post Office Lane, Beaconsfield, Bucks HP9 1QA, UK; Tel.: + 44 (0) 1494 676161 extn 3269; Fax.: + 44 (0) 1494 679331/3; e-mail: greenca@eur.perkin-elmer.com; Website: http://www. perkin-elmer.com*

### USF Elga launches an integrated range of laboratory pure water systems

Leading water purification specialist, USF Elga, have launched a complete range of water purification systems for use in laboratories. With the recent merger of USF and Elga, and the consolidation of their water purification products and technologies, USF Elga can now offer one of the widest ranges of water purification equipment available from any single supplier.

The integrated product range comprises all the technically advanced water purification systems from both USF and Elga that the laboratory sector has come to rely on. This, coupled with USF's global experience, allows USF Elga to provide laboratory customers with a single source for solutions to all their pure water requirements.

The launch of the new range, which has been consolidated and re-branded in a distinctive blue and white

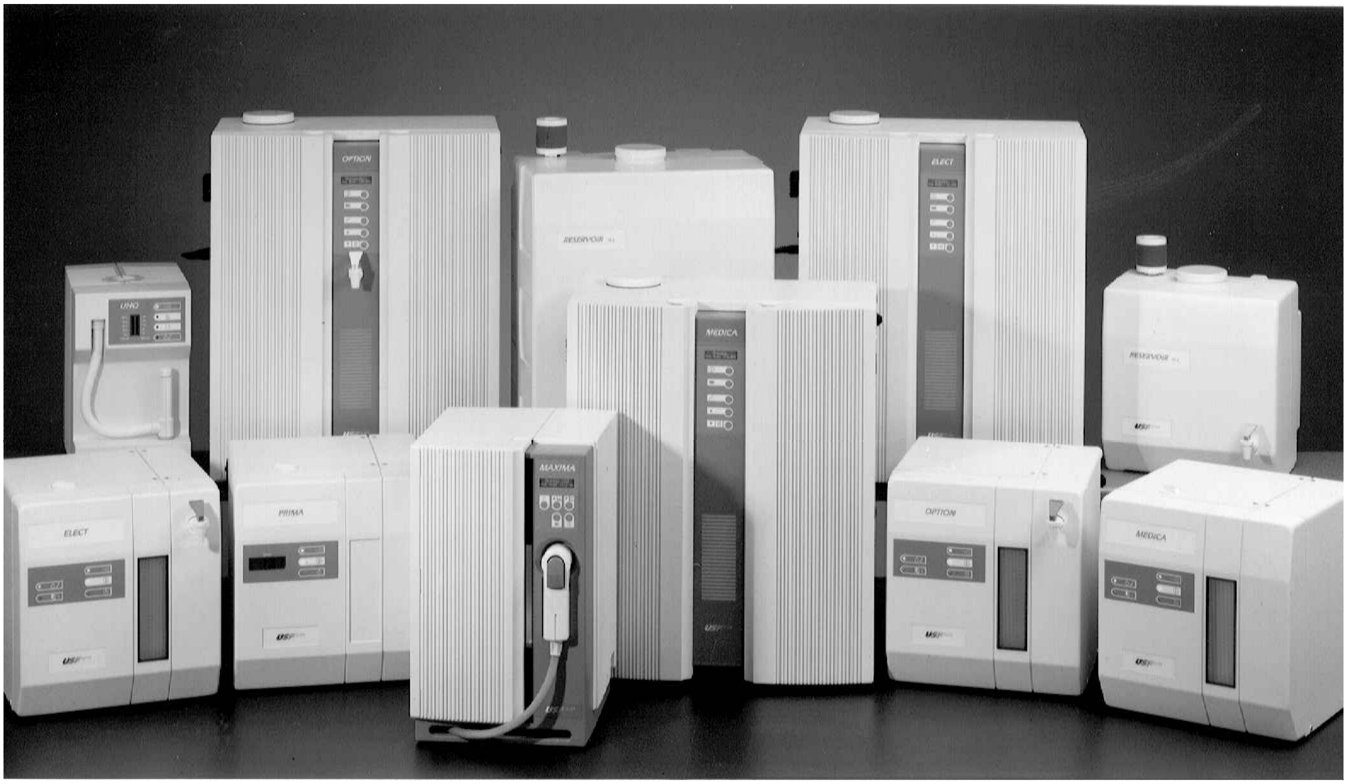

*USF Elga's integrated range of Laboratory pure water systems.*

colour scheme, marks the final stage of integration between the two laboratory water companies. The integration of the two companies means USF Elga can provide water purification systems worldwide, and support those systems with a network of over 5000 service centres.

Brad Jennings, USF Elga's International Marketing Manager comments: 'With the launch of the new product range comes the consolidation of USF and Elga in the pure water sector. The synergy of our product range, service and support gives customers a single point of reference for all their pure water requirements. The new name and appearance will ensure that customers recognize the experience and expertise behind each product, instantly. The USF Elga name will stand for excellence in the water purification products and services that we now provide to the laboratory market'.

United States Filter Corporation (NYSE/USF) is the leading global provider of commercial, industrial, municipal and residential water and wastewater treatment systems, products and services. USF is also a leading provider of outsourced water services, including the operation of water and wastewater treatment systems at customer sites. It is also actively involved in the development of privatization initiatives for municipal water treatment facilities in the United States and around the world. USF, which is based in Palm Desert, CA (USA), invites you to visit its web site at http:///www.usfilter.com or http://www.usfelga.com

With annualized revenues of over $4.5 billion, USF is the leading global provider of industrial and residential water and wastewater treatment systems, products and services. USF has developed a worldwide network of sales and service facilities in over 1700 offices and 90 manufacturing plants in 35 countries.

*For further information please contact: USF Elga; Tel.: + 44 (0) 1494 887 700; Fax.: + 44 (0) 1494 887 824.*

### HP Chemstation macros available on the internet

Geneva, Switzerland, 16 February 1999: Hewlett-Packard Europe today announced that macros for the multi-technique HP ChemStation are now available on the HP Chemical Analysis Web site at http://www.hp.com/go/chem

The macros, which are designed to help HP ChemStation customers use the software more effectively, provide shortcuts to commonly performed tasks such as the following:

- creation of post-sequence compound reports in Microsoft® Excel;
- transferring sequence files between the HP ChemStation and a server or laboratory information-management system;
- automatic e-mailing of reports; and
- editing and debugging of macros developed by the customer.

The macros are part of an expanding set of free software available on the Chemical Analysis site. They join the HP flow-calculator and method-translation software tools, which allow gas chromatographers to calculate quickly and easily pressures or flows, and transfer developed methods to GC systems using different detectors, columns or carrier gases.

*About HP and its chemical Analysis Group*

Hewlett-Packard Company is a leading global provider of computing, Internet and Intranet solutions, services, communications products and measurement solutions, all of which are recognized for excellence in quality and support. HP has 124 600 employees and had revenue of $47.1 billion in its 1998 fiscal year. Hewlett-Packard manufacturers in five European countries: Germany, UK, France, Spain and Italy.

HP's Chemical Analysis Group is a leading provider of chemical analysis measurement and information solutions, services and support, columns and supplies to scientific laboratories in industry, government and academia. The group has 3789 employees worldwide and had revenues of $946 million in its 1998 fiscal year. In Europe, the group has sales and support offices in nearly all countries, and development and manufacturing facilities in Waldbronn, Germany. The company's liquid chromatography, capillary electrophoresis and spectrophotometry products are developed in Waldbronn for worldwide markets, with design and production processes certified to the ISO 9001 international quality standard.

Information about HP chemical analysis products and services, and the company's year 2000 program can be found on the World Wide Web at http://www.hp.com/go/chem

*For more information contact: Hewlett-Packard Europe, PO Box 165, Godalming, Surrey GU7 1HP, UK; or, for press information, please contact Emma Weitkamp or Pam Pickering at HCC De Facto, Network House, Basing View, Basingstoke, Hampshire RG21 4HG, UK; Tel.: + 44 (0) 1256 842274; Fax.: + 44 (0) 1256 469308.*

### Announcements

### New food technology database launched on STN international

*Premier online service releases FROSTI database*

*Karlsruhe, Germany*: FIZ Karlsruhe, the European partner of STN International, the premier science and technology online service, has enhanced its offering of food and agriculture information, with the launch of the FROSTI database.

A vital resource for anyone wanting to keep up to date with published information on food science and technology around the world, FROSTI covers all aspects of the food and drink industry, including ingredients and process technology, quality control, microbiology, manufacturing, packaging, food chemistry, biotechnology and additives.

Produced by the Leatherhead Food Research Association (LFRA), in the UK, FROSTI contains more than 423 000 records, 95% of which include abstracts. Sources include more than 8000 scientific and technical journals published worldwide, as well as technical reports, books, bulletins, conference papers, grey literature, and US, Japanese, European, PCT and UK Patents—with coverage extending from 1972 to the present.

A key strength of the database is its currency, with major journals being abstracted and available online within two weeks of delivery. The database is updated twice weekly and SDIs (which provide automated current awareness facilities) can be run weekly.

STN International can be assessed by dialling into STN's online service using its powerful proprietary client software, STN Express with Discover! In addition, from mid-April, the service will be accessible directly over the Internet using a standard web browser (http://stnweb.-fiz-karlsruhe.de)

Note: FIZ Karlsruhe, the leading information centre for science and technology in Germany, provides various information services in printed and electronic form. In addition, FIZ Karlsruhe operates the European Service Centre of STN International, the Scientific & Technical Information Network, which offers worldwide access to bibliographic, factual and full-text databases in science and technology (more than 220 at present), among them a comprehensive cluster of the world's largest and most important patent databases. STN International is jointly operated by FIZ Karlsruhe, Chemical Abstracts Service (CAS) in Columbus, OH, and the Japan Science and Technology Corporation, Information Centre of Science and Technology (JICST) in Tokyo.

*For further information please contact: Rüdiger Mack, STN Service Centre Europe, FIZ Karlsruhe, PO Box 2465, Karlsruhe, D-76012, Germany; Tel.: + 49 7247 808 513; Fax.: + 49 7247 808 131; e-mail: rm@fiz-karlsruhe.de*

### Biotechnology business information enhanced on STN International

*Premier online service launches BIOCOMMERCE database*

*Karlsruhe, Germany*: FIZ Karlsruhe, the European partner of STN International, the premier science and technology online service, has enhanced its offering of business information for the biotechnology, chemical and pharmaceutical industries, with the launch of the BIOCOMMERCE database.

Offering comprehensive information on the business aspects of biotechnology and biological sciences worldwide, BIOCOMMERCE covers all aspects of biotechnology—including genetics, immunology, microbiology and molecular biology—as well as their applications in areas such as agriculture, brewing, chemicals, energy, foods, health care and waste treatment.

In addition, BIOCOMMERCE provides company profiles of more than 2500 companies and institutions in biotechnology, with more than 11 000 contact names.

The database includes a wealth of authoritative and timely information covering strategic alliances, mergers and acquisitions, sales and financial results, technology licensing, product news, research advances, and information on patents—as well as market data, e.g. stock offerings and share prices.

Produced by BioCommerce Data Limited, based in the UK, the database contains more than 170 000 records, including abstracts and references, and coverage extends from 1981 to the present.

BIOCOMMERCE corresponds in part to the print abstracting journal *Biocommerce Abstracts*, as well as the printed directories *The UK Biotechnology Handbook* and *The European Biotechnology Directory*. Information is drawn from more than 100 trade publications, newsletters and newspapers, and every abstract in the database includes a concise, informative summary, plus up to 30 bibliographic references. BIOCOMMERCE is updated every two weeks, and SDIs (which provide automated current awareness service facilities) can be run at the same interval.

STN International can be accessed by dialling into STN's online service using its powerful proprietary client software, STN Express with Discover! From mid-April, it will also be accessible directly over the Internet using a standard web browser (http://stnweb.fiz-karlsruhe.de).

Note: Fiz Karlsruhe, the leading information centre for science and technology in Germany, provides various information services in printed and electronic form. In addition, FIZ Karlsruhe operates the European Service Centre of STN International, the Scientific & Technical Information Network, which offers worldwide access to bibliographic, factual and full-text databases in science and technology (more than 220 at present), among them a comprehensive cluster of the world's largest and most important patent databases. STN International is jointly operated by FIZ Karlsruhe, Chemical Abstracts Service (CAS) in Columbus, OH, and the Japan Science and Technology Corporation, Information Centre of Science and Technology (JICST) in Tokyo.

*For further information please contact: Rüdiger Mack, STN Service Centre Europe, FIZ Karlsruhe, PO Box 2465, Karlsruhe, D-76012, Germany; Tel.: + 49 7247 808 513; Fax.: + 49 7247 808 131; e-mail: rm@fiz-karlsruhe.de*

**P S Analytical appoints a sales manager for Europe**

With a consistent record of sales growth in many world markets during recent years, P S Analytical is pleased to announce an appointment which centres a special responsibility for Europe.

Andrew Barker, until recently with Unicam Atomic Absorption, has taken full responsibility for the European territory with its many established distributors, to expand sales of existing products and introduce new products that are close to launch.

Andrew is an experienced salesman with experience of many analytical techniques including those relevant to PSA. 'His international sales success with his previous company will bring the correct blend of professionalism to our distribution outlets and allow us to achieve results closer to our true market potential' said Paul Stockwell, International Sales Manager.

PSA offers a range of products to determine elements that are contaminants in solid, aqueous and gaseous media. These include mercury, arsenic, selenium and antimony with detection levels as low as a few ppt. Current global regulations and legislation place increasing requirements on low levels of detection of these and similar elements. Speciation of the various elements is also a PSA speciality.

*For any communication regarding the above, please contact: Paul Stockwell at P S Analytical, Arthur House, Crayfields Industrial Estate, Main Road, Orpington, Kent BR5 3HP, UK. Tel: + 44 (0) 1689 891211; Fax: + 44 (0) 1689 896009; e-mail: pms@psanalytical.demon.co.uk; URL—http://www.psanalytical.com*

### New software and systems

**Cost-effective loop and logic control is an ideal solution for smaller processes**

Honeywell's new UMC800 Universal Multiloop Controller provides flexible and cost-effective loop and logic control for smaller single-unit processes. The UMC800 is an ideal, best-value solution for processes currently being satisfied by small integrated control systems, or several single-loop controllers and a small PLC.

By combining analogue and digital control in a single, easily implemented product, the UMC800 increases efficiency, while reducing hardware, installation and maintenance costs. It is the ideal solution for furnaces, ovens, cookers, environmental chambers, reactors, freeze dryers, extruders, and many other plants and processes.

The UMC800 has three components: a powerful controller with modular I/O (inputs/outputs) for loop and logic control; an industrially hardened operator interface with a colour display for clear process monitoring; and intuitive Control Builder configuration software for computers operating under Windows 95 or Windows NT. Control Builder is so user-friendly that, if a user has difficulty understanding function blocks, it literally draws a helpful picture.

The UMC800 controller includes a card rack supporting up to 16 input and output modules, of eight types. Modules can be mixed to satisfy the hardware requirements of specific applications. The controller's universal input cards directly accept a wide variety of sensor types, while control outputs can be current or pulse. It features advanced PID control algorithms, including autotuning and fuzzy logic overshoot protection, which optimize performance and reduce start-up times.

The UMC800 operator interface provides a wide selection of pre-formatted displays and direct access display keys to get users on-line easily and quickly. Analogue data and digital status information can be viewed clearly in multiple formats on the colour graphic LCD display. Its intuitive design reduces training needs, eliminates errors and enables efficient process management.

The UMC800's Control Builder configuration software features 'drag and drop' techniques for graphic icons, and software connections between function blocks to create custom control strategies. A large control algorithm library simplifies configuration tasks, and handles complex batch or continuous applications with ease.

All the UMC800 controller's analogue inputs are isolated and are programmed universally such that each input point of each card can have a different sensor type unlike many PLCs.

The UMC800 controller can execute up to eight loops of PID or on/off control. Accutune$^{TM}$ auto-tuning and fuzzy logic overshoot suppression are standard for all loops. Configurations support cascade, ratio, feedforward and duplex output control strategies. Separate algorithms are provided for three-position step control and carbon potential for atmosphere furnaces. Digital interface blocks are provided to allow control loop status to interact with other analogue and digital functions.

Up to eight analogue outputs may be scaled to any span between 0 and 20 mA. Up to eight time proportioning outputs are provided with a minimum on-time and minimum off-time setting to reduce unwanted cycling. Three-position step outputs are provided to adjust motor position without a position sensor. On/off and time proportioning outputs may use any digital output of any output module.

Up to four setpoint programme algorithms can be programmed, as standard. Each programmer can have up to 16 events outputs and will accomodate profiles up to 50 segments in length. Any segment may be a ramp or soak. The controller will store up to 70 profiles that are shared by the four programmers. Profiles can be developed or edited from the operator interface while the controller is on-line.

Recipes provide a method of changing up to 50 variables of the controller through a single selection. One or more of the variables may be an entire profile for a setpoint programmer selected by number.

The controller offers up to 250 function blocks and an algorithm library of more than 70 algorithm types.

*For further information please contact: Honeywell Control Systems Ltd, Honeywell House, Arlington Business Park, Bracknell, Berkshire RG12 1EB, UK. Tel.: 01344 656000; Fax.: 01344 656240. e-mail: info.centre@uk.honeywell.com*

*Honeywell Industrial Automation & Control European web site: http://europe.iac.honeywell.com*

*Honeywell Industrial Automation & Control global web site: http://www.iac.honeywell.com*

**Metrodata TiNET2.3 Software . . . the key to computer supported titration on CD ROM**

Metrodata TiNet 2.3 is a Windows™ program (version 95, 98 or NT) that comprehensively supports all aspects of titration: method development, sample data memory, real-time titration curve, documentation and saving of results; reprocessing of the measured data and exporting of results, e.g. to a LIMS.

The components of the TiNet 2.3 network are the instruments of the Titrano range as well as 713 pH meter. The 765 Dosimat, 700 Dosino and several Metrohm sample changer models are available for expansion to an automated titration system. A balance from one of the established manufacturers can also be integrated into the system. For complex problem solutions, up to 32 COM ports can be made available on your computer using a Moxa card.

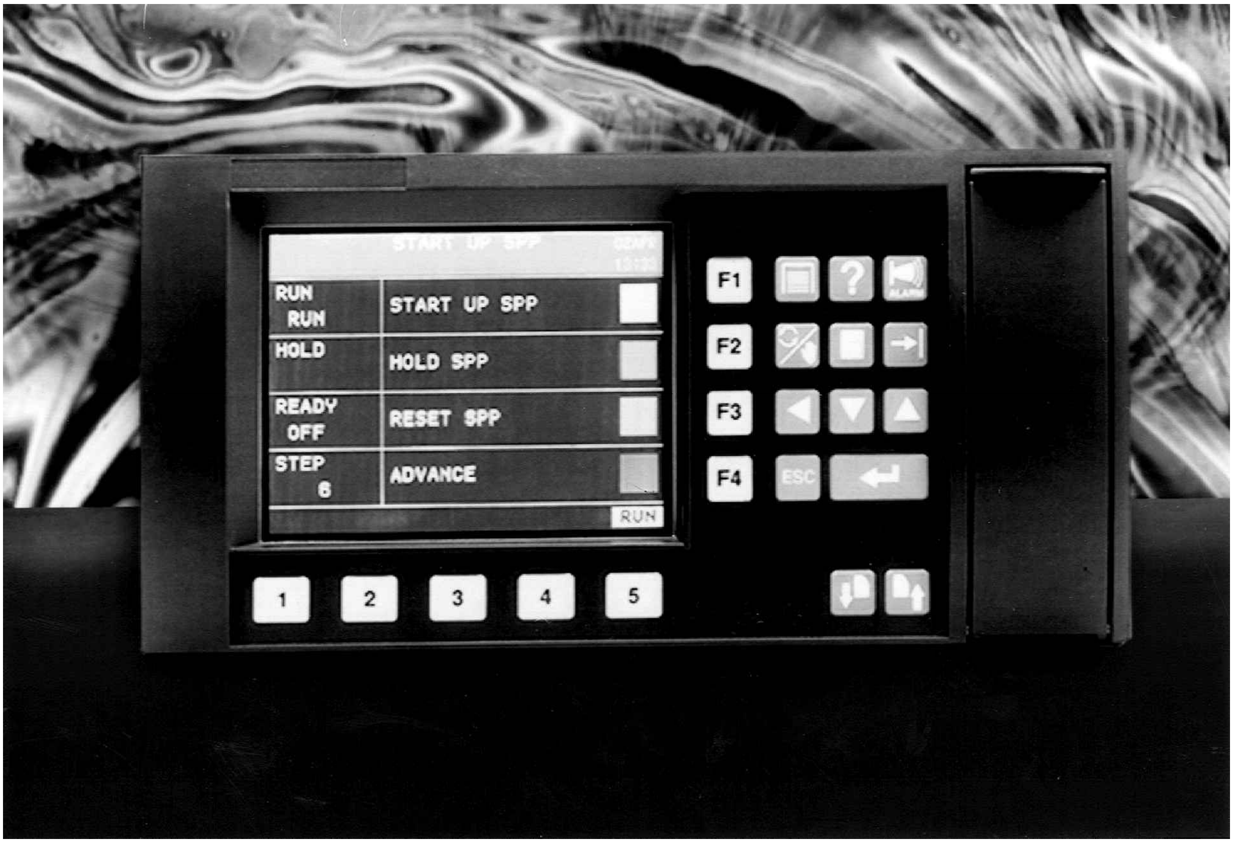

*Honeywell's UMC800 includes an industrially-hardened operator interface with a colour display for clear process monitoring.*

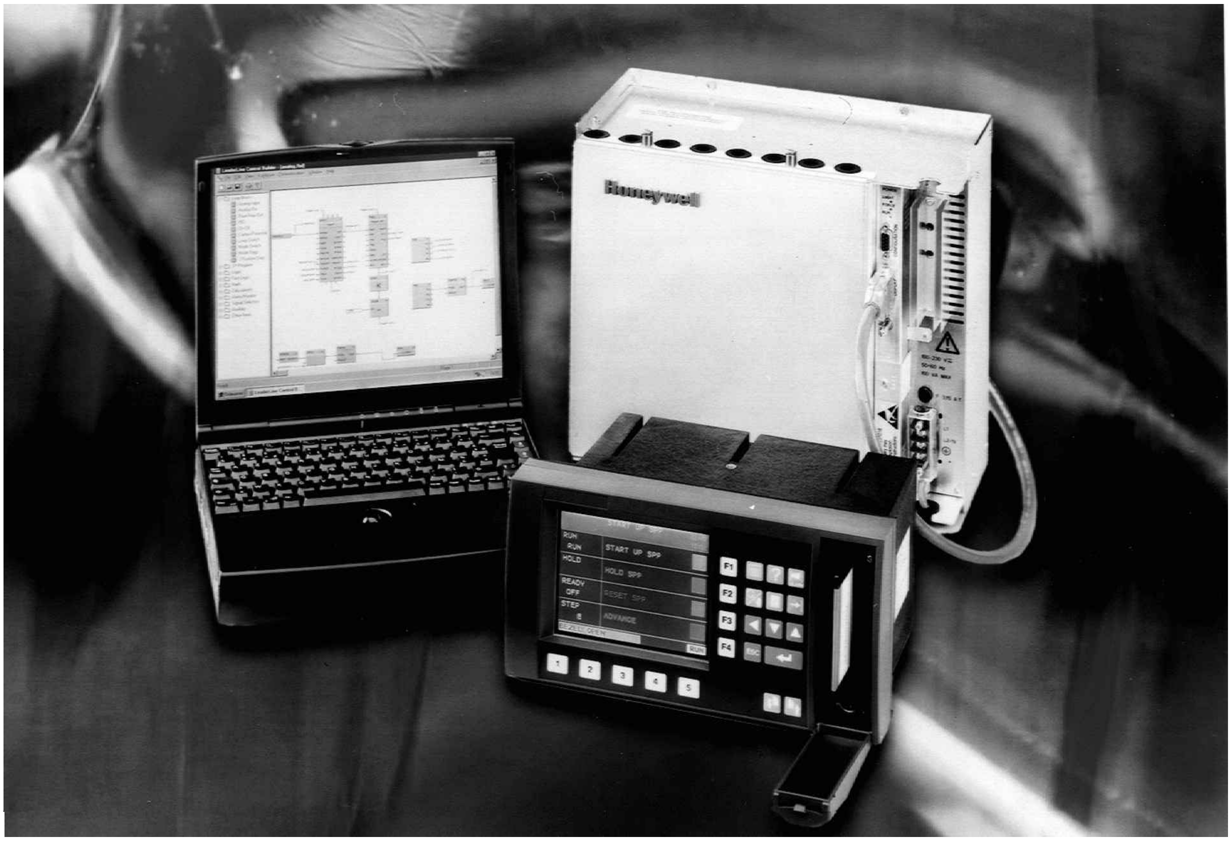

*Honeywell's UMC800 has three components: a powerful controller for loop and logic control; an operator interface with a clear colour display; and intuitive, user-friendly Control Builder configuration software.*

It is possible to assign user-specific access rights; thanks to the password protection any number of users can be registered together with their individual access rights.

Method components, e.g. titration, dosing, measuring, calculation and documentation can be linked with each other in any number and any sequence. If the titration system is operated together with a sample changer, then the freely programmable sample changer sequences are also components of the titration method. The new 748 DH sample changer with racks for 48 or 136 samples can only be controlled by TiNet 2.3.

The methods are stored on the hard disk of the PC. Backup of titration methods on storage media, e.g. diskettes, network drives or CD-ROMs presents no problems.

In addition to the enpoints found by the Titrino, the program is able to automatically evaluate special titration curves, e.g. from photometric or conductometric titrations. Evaluation according to Gran can also be carried out.

The program is available on CD-ROM in English or German. Earlier TiNet versions can be updated.

*For further information please contact Metrohm UK Ltd, Tel.: + 44 (0) 1280 824 824.*

### Announcing the release of Origin® 6.0

*Leading software for scientists and engineers raises the standards for data analysis and technical graphics in Windows®*

New: Windows® Explorer-like interface for project organization, drag and drop plotting, data masking tools, revamped GUI for comprehensive graph controls, colour mapping support, expanded graphics export capabilities, new and improved graphs, and custom toolbars.

Northampton, MA, May 1999, Microcal® Software, Inc. announces the release of Origin® version 6.0, a leading Windows®-based data analysis and technical graphics software. Known for its unsurpassed speed, Origin's new interface and expanded interactive functionality will maximize productivity by enabling scientists and engineers to produce results quickly without answering a chain of questions. With new and improved interface features, Origin 6.0 makes complex graphing and analysis fast and easy. Features include data point masking tools for controlled analysis, a new interface for customizing toolbars, a comprehensive Plot Details dialogue box for easy customization of every graphical element, and a new Project Explorer for organizing your workspace. Origin 6.0 offers expanded graphic export filters including PSD, EPS, PDF, DXF and AI, among many others. Origin 6.0 is a 32-bit product compatible with Windows® 98 and Windows NT®, and is also an OLE2 server.

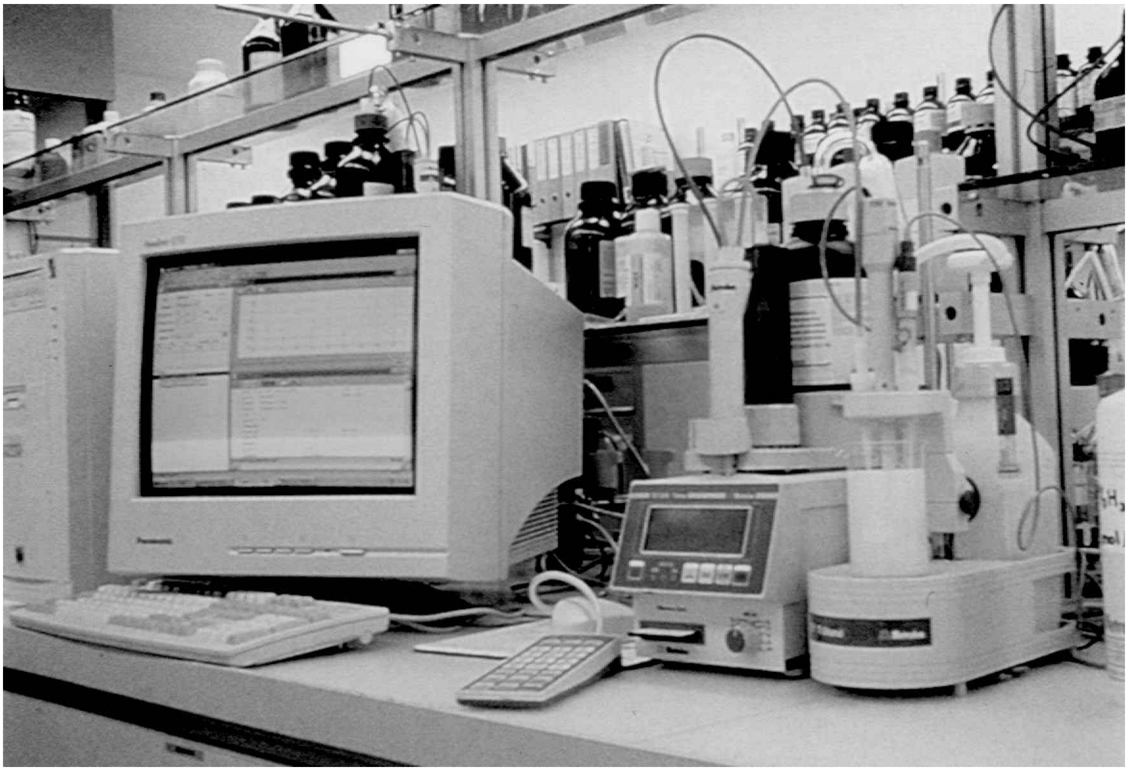

*Metrodata TiNET2.3 Software in use.*

'We have endeavoured to create the ultimate software tool to graph data and do basic to advanced analysis using statistics or mathematical functions', says Microcal Software's founder and President, Dr C. P. Yang. 'Origin has always provided unparalleled speed and flexibility. With this release all of Origin's sophisticated features are now available in an easier to use interface—one that allows both novice and advanced users the ability to create graphs and analyse their data within minutes of opening the box'.

For over 8 years Microcal Software has provided a complete solution for scientists and engineers who need to analyse, graph and professionally present data. Origin 6.0 is easier to use than ever before; even the novice user will have no problem learning to enter, graph, analyse data, and present professional results. Yet it is sophisticated enough to let advanced users automate data analysis by creating scripts with the built-in programming language. The interface incorporates the following new features.

*Organize work with the Project Explorer.* Set up an Origin project to warehouse your experimental analysis. Now keep all successive work neatly contained in the same project, using folders to file data and graphs. Just click on a folder to see the Origin workspace dynamically show its contents.

*Drag and drop plotting.* Now adding data plots to existing Origin graphs has never been easier; simply select the data in the Origin worksheet or Excel workbook, then click and drag a selection into the graph.

*Mask selected data points to focus analysis.* Use the new Mask toolbar to mask out graphically any points on the data plot. Use the tool to mask out outliers or bad data ranges that are reasonable to ignore. Once the points are masked out, any subsequent analysis performed will disregard masked points. Other tools on the toolbar allow you to change the look of the data plot, temporarily removing masked points from view.

*Access graph elements with a single dialogue box.* The new Plot Details dialogue box gives instant access to any feature of graphical data. Customization options are dynamically displayed, based on the graphic element selection within the dialogue box. Customize any data plot attribute—data points, data plots, error bars, data labels, functions, layers or the graph page—all in one interactive dialogue box.

*Expanded symbol gallery.* The new Plot Details dialogue box supplies a symbol gallery to pick from over 100 built-in geometric symbols. User-defined bitmapped symbols can be added to the gallery. Alternatively, you can choose from characters of any font installed on your computer, and have Origin increment the character for each data point.

*New colour palette library and colour mapping.* Origin now supports the use of colour mapping to represent another dimension in 2D, and 2D Contour graphs as well as 3D Surface and Contour graphs. Set up a custom colour palette to range from one colour to another, or specify colours for certain ranges. To provide a legend for the reader, an option of creating a colour scale is provided to show value ranges corresponding to colour.

*Expanded export filters*

Origin 6.0's new graphic export filters include:

| | |
|---|---|
| Adobe Illustrator | *.AI |
| Computer Graphics Metafile | *.CGM |
| Encapsulated PostScript | *.EPS |
| AutoCad Drawing Interchange | *.DXF |
| Macintosh PICT | *.PCT |
| Tag Image | *.TIF |
| Adobe Acrobat | *.PDF |
| X-Windows PixMap | *.XPM |
| Adobe Photoshop | *.PSD |
| X-Windows Dump | *.XWD |

Origin continues to support export of Windows Metafile (*.WMF), bitmap (*.BMP), JPEG (*.JPG), PCX (*.PCX) and Targa (*.TGA) files.

*New and improved graphs*

- *Vector graphs*—Origin now supports two data formats for vector graphs: the X,Y, Angle, Magnitude format, and the new X,Y X,Y format where you can simply set the tail and head coordinates. The X,Y, Angle, Magnitude format can now also be easily scaled by simply specifying a scaling factor, so that the original values do not have to be transformed.
- *Pie charts*—New Origin supports 3D pie charts, including exploded slices. You have complete control over their look—you can change the thickness of the pie slices, as well as their displacement, view angle, size and rotation of the chart.
- *Box charts*—New enhanced box charts. Now you can superimpose a column scatter graph of the data right on or next to the box chart. Further, you can place a distribution curve on the data, using any of seven standard distribution functions.

*Create custom toolbars.* Create custom toolbars to better organize the workspace. Add and remove buttons from any of Origin's existing toolbars, or create new toolbars containing a combination of buttons from Origin's existing toolbars.

*Pricing information*

Origin version 6.0 is available for $595. Current US Origin users can upgrade from version 5.0 to version 6.0 for $199, or from any earlier version for $299.00. All upgrades include the 2D and 3D graphing capabilities, as well as all new manuals including a new Tutorial Manual.

*System requirements*

Microsoft Windows 95 or later, or Windows NT® version 4.0 or later, 486/DX or higher processor, 16 megabytes (MB) of RAM (32 MB recommended). A CD-ROM drive, 18 MB of available hard drive space.

*About the Company*

Microcal Software develops products that are easy to use, yet have the power and versatility to provide a complete solution for the needs of scientists and engineers. Since the release of Origin in 1991, the company has sold over 35 000 copies of Origin, and has evolved as a leading player in the technical graphics and data analysis market.

*Evaluation copy*

For more information on receiving an evaluation copy, please call Amanda Cottrell at (413) 586-2013 ext. 148.

*For more information on Microcal Software's other products, visit http://www.microcal.com*

### A breakthrough in science from Bio-Tek

With the introduction of their new range of Microplate spectrophotometers, Bio-Tek Instruments have achieved real innovation in breakthrough science. The completely new MicroTek DS merges the throughput and convenience of microplate format with the performance of a spectrophotometer. Conventional EIAs at virtually any wavelength, quantitate nucleic acids in the lower UV range or automatically run spectral scans for peak absorbance measurements. Among the MicroTek DS's many features are custom diffraction grating, spectral scanning, multiple plate formats and pre-defined route analysis.

The MicroTek DSI takes microplate spectrophotometry one leap further with its unique four-zone incubation design and multichannel reading speed. The 200–999 nm range encompasses low-UV and near-IR measurements, while the 5 nm bandpass ensures precise measurements for nucleic acid quantitation. With improved reproducibility in enzyme kinetic reactions, measurements in intervals as short as 8 sec and the ability to report kinetic rate, $R^2$ or onset time without needing a PC, the MicroTek DSI is a superior tool for high-speed kinetic measurements. For HTS needs, MicroTek DSI interfaces to many robotic workstations and automated systems.

With a range of accessories and data analysis software, the new Bio-Tek range of Microplate spectrophotometers assists today's scientist in breaking through barriers of traditional spectrophotometry to obtain results quickly, accurately and reliably.

*Bio-Tek Instruments, 8 Marlin House, Croxley Business Park, Watford, Hertfordshire WD1 8TA, UK. Tel.: + 44 (0) 1923 691300. Fax.: + 44 (0) 1923 691301. URL—http://www.biotek-kontron.com*

### New microbalance software from CI

CI introduces Windows software to bring new ease of operation and cost efficiency to users of its microbalances.

Offering real-time display of weight changes with 0.1 µg resolution, and temperature changes with 0.3°C resolution, LabWeigh allows the user to fine tune experiments for optimum results or save time by aborting unproductive runs.

LabWeigh enables weight and temperature, as well as rate of change, to be logged at specified intervals over periods from 1 min to 60 days, to complement CI's robust microbalance solutions for long-term experiments. Its easy to use facilities simplify calibration, set up, and plotting of results on customisable graphs. Results of current or previous experiments can be examined with the advantages of zoom and cursor readout features.

CI's choice of the Windows environment allows results to be downloaded to popular spreadsheet or wordprocessor packages for rapid report generation, and also allows the PC to monitor experiments concurrently with performing other tasks.

Offering outstanding versatility and price/performance for measuring any force which can be related to weight, CI's microbalances operate on the electromechanical comparator principle to command a worldwide market both in fundamental materials research, and in oil, nuclear, pharmaceutical and other industries.

They are based on a modular concept, and can be supplied with all accessories for adaptation to specific requirements or integration into users' own system designs. For example, CI can supply a range of programmable furnaces for thermogravimetric applications, and will use its considerable applications experience to assist customers in tailoring total measurement solutions.

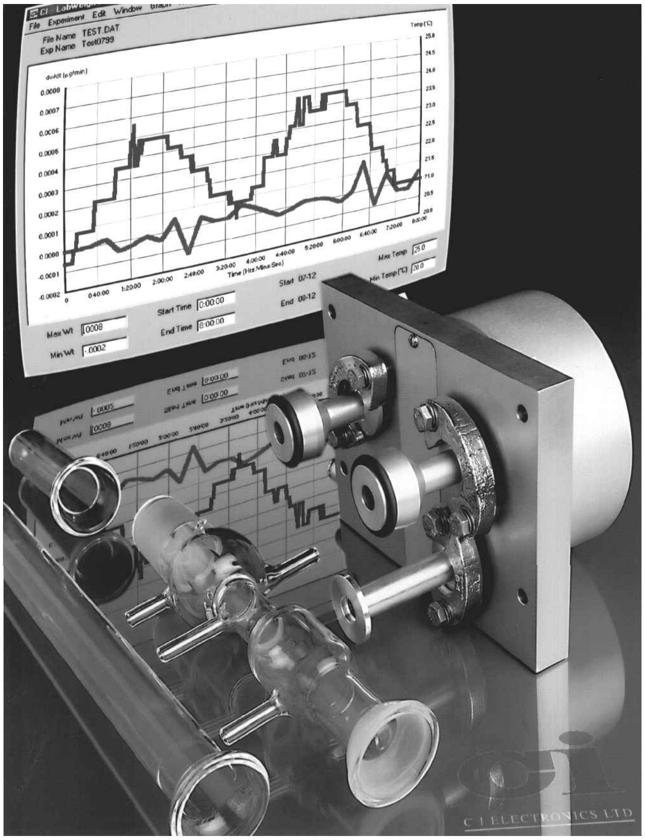

*CI introduces Windows software to bring new ease of operation and cost-efficiency to users of its microbalances.*

*For CI's latest microbalance catalogue or further information contact Janet Cranage, C. I. Electronics Limited, Brunel Road, Churchfields, Salisbury, Wiltshire SP2 7PX, UK. Tel.: + 44 (0) 1722 336938; Fax: + 44 (0) 1722 323222; e-mail: admin@cielec.com; Internet: www.cielec.com.*

### New software and systems/News

### HP announces strategic realignment to create two companies

*Platt to lead realignment as HP Chairman, President and CEO; Barnholt named CEO of New Measurement Company; search begins for CEO of New Computing and Imaging Company*

*Palo Alto, CA, 2 March 1999:* Hewlett-Packard Company Chairman, President and Chief Executive Officer Lewis E. Platt today announced plans for a strategic realignment of Hewlett-Packard company that will create two independent companies.

'We are taking this action to sharpen the strategic focus of our businesses, improve ... agility and increase ... responsiveness to customers and partners', said Platt. 'This will offer exciting opportunities for our employees and will enhance the two new companies' growth and earnings potential'.

The HP Board of Directors approved pursuing the realignment plan at a special meeting this morning. Under the plan, HP stockholders will hold shares in each company.

'We are creating two distinct and strategically focused enterprises, one focused on the measurement business, the other on the computing and imaging businesses', said Platt. 'These new companies will be financially strong and independently managed, each with its own board of directors and its own central research-and-development organization. Both companies will be able to better focus on growth in their individual markets. They will be well-positioned to build value for their stockholders, customers and employees'.

The new measurement company will comprise Hewlett-Packard Company's industry-leading test-and-measurement, components, chemical-analysis and medical businesses. These businesses represented $7.6 billion of HP's total revenue of $47.1 billion in fiscal 1998. With leading positions in multiple market segments, the technology-based company will be focused on high-growth opportunities, e.g. communications and life sciences. A name has not yet been selected for the new measurement company.

Edward W. (Ned) Barnholt, currently HP Executive Vice President and General Manager of the Measurement Organization, has been named Chief Executive Officer of the new measurement company. 'I've asked Ned to take on this critical leadership role', said Platt. 'He has the vision, experience and enthusiasm to position the company for accelerated growth and success'.

Hewlett-Packard Company is considering an initial public offering (IPO) for approximately 15% of the measurement company's outstanding shares by year-end. This offering would be the largest technology IPO in Silicon Valley history. Subsequent to the IPO, HP would expect to distribute the remaining shares to its stockholders in a tax-free transaction.

The new computing and imaging company will continue to operate under the Hewlett-Packard name, and will include all of HP's enterprise computing systems, software and services, personal computer, and printing and imaging solutions businesses. These businesses will operate more autonomously and will adapt quickly to changing market needs.

Platt, who drove the plan to create two companies, will continue as HP Chairman, President and Chief Executive Officer, and will manage the transformation until the separation is completed. Platt and HP board members John B. Fery, Sam Ginn and Richard A. Hackborn will constitute a special committee of the HP Board of Directors what will conduct a search for a Chief Executive Officer for the new computing and imaging company. Ginn will chair the committee.

'The two new companies we're creating compete in fast-moving, highly competitive markets', said Platt. 'This realignment positions each new company to deliver enhanced growth in revenue and earnings'.

### *The new measurement company*

The new measurement company, which includes the original HP businesses, will be one of the world's premier technology-based companies focusing on high-growth opportunities, e.g. communications and life sciences. Its products and services are used widely to design, build and manage today's most advanced communications networks, including the Internet. In addition, the company has leadership positions in fiber-optic, wireless and optoelectronics components. The company is a leader in certain segments of the medical-product and chemical-analysis markets, and will be pursuing new opportunities for growth in healthcare and biosciences.

'We are building an exciting new company based on the strong heritage of HP technology, a culture of innovation and the excellent people throughout our organization', said Barnholt. 'We will focus our strength on high-growth opportunities and extensions into new markets'.

### *The new computing and imaging company*

'Our computing and imaging company will be focused on providing unique solutions for the computer, printer and information-services markets', said Platt. Under the banner of the recently articulated e-services initiative, all of the computing and imaging businesses will capitalize on the opportunities in the second chapter of the Internet, the mass proliferation of electronic services.

'The first chapter of the Internet was about access to information and self-service transactions', said Platt. 'In the second chapter, modular electronic services will proliferate. HP will provide the fundamental innovations and solutions that will make it easier for companies to build and deploy e-services, including enabling technologies that allow these services to interact with and leverage each other. One of my top priorities is to drive the implementation of this strategy. We're confident that this will create exciting growth opportunities for our customers and for HP'.

In addition to delivering solutions with the e-service initiative, the computing and imaging businesses will continue to develop products for their individual markets. The businesses include the following:

- laserjet solutions group;
- inkjet products group;
- enterprise computing solutions organization; and
- personal systems group.

### *About the realignment*

Hewlett-Packard company does not expect any significant overall workforce reductions as a result of the realignment. 'This announcement is not about major short-term cost reductions', said Robert P. Wayman, HP executive Vice President and Chief Financial Officer. 'Where we need to realign our workforce to support the new companies, we will manage this, as we have in the past, through our traditional approaches, such as redeployment, flex-force adjustments and attrition'.

Hewlett-Packard Company will seek any required tax and regulatory approvals worldwide and expects to complete the realignment during the first half of next year.

The investment banking firms of Morgan Stanley Dean Witter and Goldman, Sachs & Co., are serving as advisors to Hewlett-Packard Company.

### *About HP*

Hewlett-Packard Company is a leading global provider of computing, Internet and intranet solutions, services, communications products and measurement solutions, all of which are recognized for excellence in quality and support. HP has 122 800 employees and had revenue of $47.1 billion in its 1998 fiscal year.

Information about HP, its products and the company's Year 2000 Program can be found on the World Wide Web at http://www.hp.com.

This news release contains forward-looking statements, based on current expectations, that involve risks and uncertainties that could cause results of Hewlett-Packard company and the measurement company to differ materially from management's current expectations. These risks include: the ability of HP successfully to manage and complete the realignment process, including the ability to retain and motivate key employees; the potential for business disruption; risks relating to the worldwide allocation of assets and people between the two companies during the process; and risks that the IPO of the new measurement company may not be completed in a timely manner or at all. In addition, other risks that HP faces in running its operations include: the timely development, production and acceptance of new products and services and their feature sets; the challenge of managing asset levels, including inventory; the flow of products into third-party distribution channels; the difficulty of keeping expense growth at modest levels while increasing revenues; the impact on customers and suppliers as they prepare for the year 2000; and other risks detailed from time to time in Hewlett-Packard's securities and exchange Commission reports, including but not limited to the annual report on Form 10-K for the year ended 31 October 1998 and the quarterly report on form 10-Q for the fiscal quarter ended 31 January 1999.

*For more information contact: Hewlett-Packard Europe, PO Box 165, Godalming, Surrey GU7 1HP, UK; or for press information, please contact: Emma Weitkamp or Pam Pickering at HCC de Facto, Network House, Basing View, Basingstoke, Hampshire RG21 4HG, UK; Tel.: + 44 (0) 1256 842274; Fax.: + 44 (0) 1256 469308.*

### MDS Harris makes breakthrough discovery in bioanalytical laboratory

*Lincoln, NE—20 May 1999:* The bioanalytical laboratory at MDS Harris has made a breakthrough discovery that will greatly speed the analysis of the compound ethinyl oestradiol.

Two MDS Harris scientists, Lee Zhu, PhD and Naidong Weng, PhD, recently discovered how to validate ethinyl oestradiol using an LC/MS/MS instrument. Until this breakthrough, the compound had to be validated using a GC/MS instrument, which is a much slower process.

'This inovation is so unique, we are going to protect this intellectual property using appropriate legal processes,' said Jim Hulse, PhD, senior vice president, MDS Harris.

As a result of this discovery, MDS Harris is now the first and only bioanalytical laboratory to offer ethinyl oestradiol at a limit of quantitiation of 5 pg/ml on the LC/MS/MS. This translates into a sample run time of only four. For a clinical study with 2000 samples, preliminary data can now be reported in only 10 working days.

MDS Harris has long been recognized for its leadership in the application of LC/MS/MS technology and for its dedicated team of bioanalytical scientists.

'Our scientists have backgrounds in chemistry, biochemistry, analytical chemistry, immunochemistry, biological sciences, mass spectroscopy, pharmacology and toxicology,' said Hulse. 'Our clients count on the specialized experience and extensive backgrounds of our scientists to develop innovative techniques and robust, sensitive assays.'

The new discovery from Zhu and Weng is now counted among the nearly 400 methods developed by MDS Harris to expedite bioanalytical research.

The company's bioanalytical services include method development and validation, high-volume sample processing, high-volume assay capabilities and applications, special testing capabilities, surrogate (biochemical) markers, and ongoing data transmission.

MSD Harris, founded in 1993, is headquartered in Lincoln, NE, and has grown to become a leading international clinical research organization serving the needs of the pharmaceutical and biotechnology industries. MDS Harris provides Phase I, II, III and IV clinical research, SMO, bioanalytical, pharmacokinetic, statistical and data management services from sites throughout North America, Europe, Africa and Asia.

MDS (MHG.A, MHG.B) is a Canadian-based, international health and life sciences company. Its core purpose is to make a distinctive contribution to the health and well-being of people. MDS employs more than 7000 highly skilled people at its global operations in Canada and the USA, as well as in Northern Ireland, England, Belgium, Switzerland, Germany, South Africa, Japan, Taiwan and China. The core businesses of MDS include: the production and distribution of diagnostic and therapeutic radioisotopes; the provision of laboratory information and management, and patented laboratory automation and software; drug discovery and development services for the world's biotechnology and pharmaceutical industries; manufacturing of ultra-trace detection equipment; and medical and hospital supplies and inventory management systems. Detailed information about the company is available on the MDS Web site at http://www.mdsintl.com or by calling 1-888-MDS-7222.

*For further information, please contact: Angie Thorell, MDS Harris, 621 Rose Street, Lincoln, NE 68501, USA. Tel.: + 1(402) 476-2811, www.mdsharris.com*

### Intranet option for *Analytical Abstracts*

*Analytical Abstracts* the abstracting service designed to meet analytical chemistry information needs, is now available through Dialog OnDisc. Delivered on CD-ROM, *Dialog OnDisc Analytical Abstracts* can be used over corporate Intranets, allowing access from every desktop by means of local web browsers. *Dialog@site* software makes this possible.

Dialog OnDisc Analytical Abstracts can also be used standalone, or loaded onto a conventional LAN/WAN. The database is Windows, DOS and Mac compatible. Monthly updates are included in the cost of a years subscription, which is $1695 for standalone. (The usual network supplements apply.) More than 1300 new citations are added in each monthly update.

The specialized analyte/matrix/concept indexing features of *Analytical Abstracts*, and the database's extensive coverage of experimental detail, make this a vital tool for both theoretical and practical analytical scientists. Coverage goes back to 1980.

*Analytical Abstracts* is also available online from DataStar, Dialog, FIZ Chemie, Orbit, STN and ChemWeb, and on CD-ROM from SilverPlatter. *Analytical Abstracts* is produced by the Royal Society of Chemistry.

The Royal Society of Chemistry is a learned and professional society with a world-wide membership of 46,000. It has as its main objective the advancement of the science of chemistry and its applications, and the maintenance of high standards of competence and integrity among practising chemists. The Society has been involved with the publication of scientific literature since 1841. Surplus generated from sales funds the promotion of chemistry.

*For further information, or to request a free 30-day trial of* Dialog OnDisc Analytical Abstracts, *Please contact: Claire Lucas, Royal Society of Chemistry, Thomas Graham House, Science Park, Milton Road, Cambridge, CB4 0WF, UK. Tel: +44 (0) 1223 432300, Fax: +44 (0) 1223 423429, e-mail: sales@ rsc.org*

### News/Forthcoming event

The International Ion Chromatography Symposium 2000 will be held in Nice, France, during 11–14 September 2000.

*For more information, contact Janet Strimaitis, Century International, P.O. Box 493, Medfield, MA 02052-0493, USA. Tel.: +1 508/359-8777; Fax: +1 508/359-8778; www.icsymposium.com; e-mail: century@fiam.net*

